# Fractionation of Whey Protein Isolate with Supercritical Carbon Dioxide—Process Modeling and Cost Estimation

**DOI:** 10.3390/ijms13010240

**Published:** 2011-12-27

**Authors:** Alexandra L. Yver, Laetitia M. Bonnaillie, Winnie Yee, Andrew McAloon, Peggy M. Tomasula

**Affiliations:** 1 Ecole Nationale Supérieure des Ingénieurs en Arts Chimiques Et Technologiques, 4, allée Emile Monso, 31030 Toulouse, France; E-Mail: Alexandra.yver@ensiacet.fr; 2 Dairy & Functional Foods Research Unit, Eastern Regional Research Center, Agricultural Research Service, U.S. Department of Agriculture, 600 East Mermaid Lane, Wyndmoor, PA 19038, USA; E-Mails: Winnie.yee@ars.usda.gov (W.Y.); Andrew.mcaloon@ars.usda.gov (A.M.); Peggy.tomasula@ars.usda.gov (P.M.T.)

**Keywords:** supercritical carbon dioxide, fractionation, whey proteins, alpha-lactalbumin, beta-lactoglobulin

## Abstract

An economical and environmentally friendly whey protein fractionation process was developed using supercritical carbon dioxide (sCO_2_) as an acid to produce enriched fractions of α-lactalbumin (α-LA) and β-lactoglobulin (β-LG) from a commercial whey protein isolate (WPI) containing 20% α-LA and 55% β-LG, through selective precipitation of α-LA. Pilot-scale experiments were performed around the optimal parameter range (T = 60 to 65 °C, P = 8 to 31 MPa, C = 5 to 15% (w/w) WPI) to quantify the recovery rates of the individual proteins and the compositions of both fractions as a function of processing conditions. Mass balances were calculated in a process flow-sheet to design a large-scale, semi-continuous process model using SuperproDesigner® software. Total startup and production costs were estimated as a function of processing parameters, product yield and purity. Temperature, T, pressure, P, and concentration, C, showed conflicting effects on equipment costs and the individual precipitation rates of the two proteins, affecting the quantity, quality, and production cost of the fractions considerably. The highest α-LA purity, 61%, with 80% α-LA recovery in the solid fraction, was obtained at T = 60 °C, C = 5% WPI, P = 8.3 MPa, with a production cost of $8.65 per kilogram of WPI treated. The most profitable conditions resulted in 57%-pure α-LA, with 71% α-LA recovery in the solid fraction and 89% β-LG recovery in the soluble fraction, and production cost of $5.43 per kilogram of WPI treated at T = 62 °C, C = 10% WPI and P = 5.5 MPa. The two fractions are ready-to-use, new food ingredients with a pH of 6.7 and contain no residual acid or chemical contaminants.

## 1. Introduction

Bovine whey, containing approximately 1% protein, is separated from milk during cheese production processes and is concentrated to make whey protein concentrates (WPC) or whey protein isolates (WPI) depending on the desired protein purity. WPC contains from 35 to 89% proteins and WPI more than 90% [1]. WPI contains more than seven different kinds of proteins, including more than 50% β-Lactoglobulin (β-LG) and approximately 20–25% α-Lactalbumin (α-LA) [2]. Several minor whey proteins such as immunoglobulins, lactoferrin and bovine serum albumin, and casein and fragmented casein residues are also present in smaller quantities, as well as glycomacropeptide (GMP) in WPI made from sweet whey [1].

The two main proteins of WPI, α-LA and β-LG, have different physical and functional properties, such as temperature- and pH-driven conformational changes, and resulting aggregation and loss of solubility [1]. The differences in pH and thermal sensitivity can be exploited in the extraction and separation of these two proteins. α-LA aggregates and precipitates at a different set of pH and temperature from β-LG, thus either α-LA or β-LG may be selectively precipitated under careful operating conditions while the other protein remains mostly soluble [3–7]. The α-LA-enriched fraction has been proposed for use in infant formulae and for children’s foods, as well as seniors [8–11], while the β-LG-enriched fraction may be used in nutritional drinks or to enhance gel strength in foods. Whey proteins are high in branched chain amino acids, essential amino acids and leucine [12,13] which may be used to fortify foods or in the creation of foods that target specific nutritional needs.

The precipitation of various food proteins through combined thermal and pH destabilization of one or more of the proteins is a well-known fractionation method that usually employs strong acids such as hydrochloric or citric acid [14,15]. The mild acidic and anti-solvent properties of liquid and supercritical CO_2_ (sCO_2_) have been under investigation as an alternative for the separation and purification of food proteins. sCO_2_ has demonstrated strong interactions with various proteins both in aqueous [16,17] and organic solutions [18], triggering the formation of protein particles and/or the precipitation of select proteins out of solution via acidification or anti-solvent effects. These phenomena have been utilized to extract, isolate or purify diverse types of proteins such as soy proteins [19–23], fish proteins [24,25], fruit proteins [26], porcine insulin [27] or model protein [28].

In dairy research, the solubility of sCO_2_ in milk and diverse effects of sCO_2_ on the structure and pH of milk have been characterized [29–31]. sCO_2_ lowers the pH of milk below pH 5 and greatly destabilizes casein proteins to precipitate at temperatures around 35–40 °C [32–36]. Some of the uses for sCO_2_-precipitated casein (CO_2_-Casein) include the manufacture of edible films and packaging with reduced water-solubility compared to sodium- or calcium-caseinate films [37–40].

In previous studies [33,41], we demonstrated the use of sCO_2_ to fractionate whey protein concentrates and whey protein isolates into a powdered fraction enriched with α-LA and a soluble fraction containing β-LG and GMP [42]. As sCO_2_ was mixed and dissolved in water, carbonic acid was produced and decreased the pH of the whey protein solutions. The final pH was controlled by the solubility of the gas, which depended on sCO_2−_ pressure via thermodynamic equilibrium [43]. At temperatures of 60–65 °C and between pH 4.2 and 5.0, we showed that α-LA progressively and selectively precipitated along with most of the minor whey proteins, while β-LG and GMP remained mostly soluble [14], (unpublished results).

The main advantages of the sCO_2_ whey protein fractionation process are that CO_2_ evolves when the protein fractions are extracted from the reactor and depressurized to atmospheric pressure, leaving clean protein fractions that contain no residual chemicals and are ready to use (pH 6), thereby removing the need for post-treatment and purification steps. CO_2_ can be collected and recycled back into to the reactor, providing an environmentally-friendly process. In addition, the sCO_2_ process is easy to scale-up and can handle concentrated whey protein solutions, thus enabling high production rates of the two enriched protein fractions. The main unknown is whether the sCO_2_ whey protein fractionation process is commercially affordable, considering that costly state-of-the-art equipment such as large, high-pressure stirred reactors, high-speed centrifuges, and spray-dryers will be needed. This study focused on designing a semi-continuous, commercial-scale model of our pilot-scale sCO_2_ process using experimental results that cover the entire effective range of operating parameters (T, P, C) to examine relationships between products quantity (yield), quality (purity), and the total costs of facility, production and equipment, for the manufacture of new affordable whey protein fractions for use as nutritious and health-promoting food and drink ingredients.

## 2. Materials and Methods

### 2.1. Materials

Commercial spray-dried WPI from cheese whey, Provon 190, was purchased from Glanbia Nutritionals Inc. (Richfield, ID, USA) and contained 90.1% (w/w) protein, with 3.6% moisture, 2.9% ash, and lactose and fat. The approximate protein composition as determined with a combination of electrophoresis and chromatography methods [42,44] was: 55% β-LG, 20% α-LA, 18% GMP and 7% minor whey proteins (immunoglobulins, bovine serum albumin, lactoferrin, and casein fragments).

WPI solutions with concentrations, C_WPI_ = 50, 100 or 150 g/L (5, 10 or 15 wt %) were prepared using de-ionized water produced with a Milli-Q Synthesis water purification system (Millipore, Billerica, MA, USA). Liquid carbon dioxide (CO_2_) tanks with an eductor tube were purchased from GTS-Welco (Allentown, PA, USA).

### 2.2. Pilot-Scale Process Setup and Experimental Protocol

**Figure 1:**
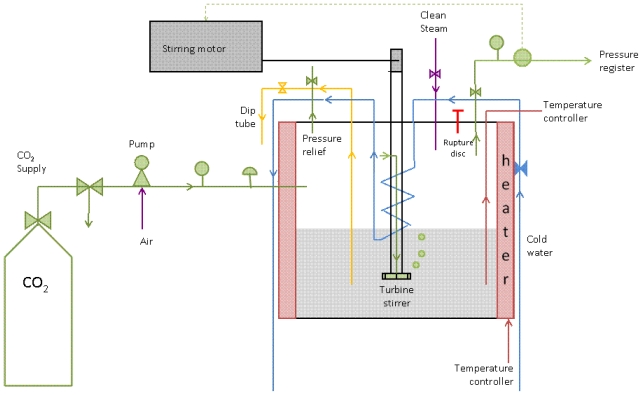
Schematic of the pilot-scale sCO_2_ protein fractionation process.

A pilot-scale batch process to fractionate the proteins of WPI with sCO_2_ was adapted from previous work by Tomasula *et al.* [33,41,45,46]. A 1-L stainless-steel, high pressure reactor from Autoclave Engineers (Erie, PA, USA), with bolted closure, floor stand, MAG 075 MagneDrive stirring assembly, and a maximum working pressure of 38 MPa was used to saturate 700-mL WPI solutions with supercritical CO_2_ (sCO_2_), as shown in Figure 1. Various accessories were included or added for control of the temperature, pressure, sampling, and safety of operation.

Liquid CO_2_ was pumped into the reactor and pressurized to supercritical conditions with an air-driven liquid pump (Haskel, Huntington Beach, CA, USA). The solution and sCO_2_ were mixed with a turbine impeller mounted on a MAG075 MagneDrive stirrer (Autoclave Engineers, Erie, PA, USA) set to 1000-rpm. A turbine impeller allowed fast (3 to 7 min) thermodynamic saturation of the liquid with CO_2_ through the dispersion and re-circulation of CO_2_ bubbles taken from the reactor’s headspace into the protein solution. The reactor was heated slowly to the desired temperature, T_R_ = 60 to 65 °C, with an electric heating mantel (Autoclave Engineers, Erie, PA) and the solution temperature was measured with a thermocouple dipped in a well inside the reactor and recorded on a computer with a programmable PID temperature-controller (Cole-Parmer, Vernon Hills, IL, USA). The reactor was pressurized with liquid CO_2_ to 5.5 MPa before heating, then additional CO_2_ was pumped into the warm reactor to reach the desired pressure, P = 8.3 or 31 MPa. Steady-state for T and P was monitored with the temperature-controller, pressure transducer and LabView software (National Instruments Corp., Austin, TX, USA) during the duration of the experiment (2 hours). After treatment, the protein mixture was extracted through a dip-tube, using the pressure inside the reactor as the driving force. During depressurization, vaporization of dissolved CO_2_ cooled the sample and produced dense, white foam. After foam collapse, pH of the sample returned to ~6.0. The sCO_2_-treated protein mixture was centrifuged at 10,000 *g* with a Sorvall RC-5B centrifuge with a SA-600 or SS-34 rotor, then the solid and liquid fractions were separated and lyophilized. The reactor base was sterilized with steam then thoroughly washed between runs. Each experiment was replicated with 2 to 4 repeats and data were analyzed using ANOVA on MS Excel 2003 software (Microsoft, Redmond, WA, USA).

### 2.3. pH Determination

In aqueous solutions, CO_2_ produces carbonic acid when dissolved, lowering the pH as a function of C_WPI_, P and T according to thermodynamic equilibrium [43] and buffering effects from the whey proteins. The pH of WPI solutions saturated with CO_2_ as a function of C_WPI_ and P was extensively measured, then calibrated for calculation as a function of C_WPI_ and P (unpublished results). Briefly, the pH of WPI solutions ranging from 10 to 280 g/L was measured at 60 °C between 0.5 and 13.8 MPa with a high pressure probe resistant to 13.8 MPa (Innovative Sensors, Inc., Anaheim, CA, USA), then modeled as a function of P and C_WPI_ with Equations 1 and 2 in order to calculate pH values at higher pressures:

(1) $$\text{pH}=-0.25\;\text{ln P}+\text{f}({\text{C}}_{\text{WPI}})$$

with P in psi, and

(2) $$\text{f}({\text{C}}_{\text{WPI}})=3.73\times 10^{-5}\;{{\text{C}}_{\text{WPI}}}^{3}-2.59\times 10^{-3}\;{{\text{C}}_{\text{WPI}}}^{2}+7.33\times 10^{-2}\;{\text{C}}_{\text{WPI}}+6.16$$

valid for C_WPI_ = 1 to 28 wt % WPI.

### 2.4. Yield and Compositions of the Fractions

The aggregate fraction yield, Y, was determined by mass difference as the ratio of solid material in the dried precipitate to the initial amount of WPI powder.

Individual recovery of protein k in the aggregate fraction, rec_k_, was calculated as the mass ratio of protein k recovered in the precipitate, divided by the amount of protein k in the initial WPI solution:

(3) $${\text{rec}}_{\text{k}}=({\text{x}}_{\text{k},\text{agg}}\times {\text{m}}_{\text{agg}})/({\text{x}}_{\text{k},\text{WPI}}\times {\text{m}}_{\text{WPI}})={\text{x}}_{\text{k},\text{agg}}\times \text{Y}/{\text{x}}_{\text{k},\text{WPI}}$$

where m_agg_ and m_WPI_ are the total mass of precipitate (aggregate) and WPI powder, respectively; and x_k,agg_ and x_k,WPI_ are the mass fractions of protein k in the aggregate fraction and the initial WPI powder, respectively, as determined via compositional analyses.

The protein distribution in both fractions was determined using sodium-dodecyl-sulfate polyacrylamide gel electrophoresis (SDS-PAGE) on a Phast System (Pharmacia, Piscataway, NJ) with 20% acrylamide homogeneous gels and 8 lanes. Samples from all the solid and liquid fractions were prepared similarly to the method of Parris *et al.* [44], using 7 wt % protein into a 2.5% SDS solution. Gel treatment included staining of protein bands with Coomassie brilliant blue dye for 20 min, and de-staining for 4 hours. Location, size and intensity of the protein bands were characterized with ImageQuaNT^TM^ software (Molecular Dynamics, Inc., Sunnyvale, CA, USA). The composition of the protein fractions was obtained using the software’s automated ‘volume %’ calculation for each protein band (the color intensity integrated over the surface area of the band) and corrected with a calibration curve.

Because GMP was not visible on SDS-PAGE gels, several samples were also analyzed with high-pressure liquid chromatography (HPLC) to measure GMP contents and adjust compositions accordingly. HPLC was run on a 250 mm long C4 column with 3.5 μm particles and 3 × 10^−8^ pores, with acetonitrile solvent containing 0.1% TFA at a flow rate of 0.8 mL/min, and UV detection set at 214 nm wavelength [42]. Protein solutions were diluted to 1 wt % concentration.

### 2.5. Commercial-Scale Process Design and Modeling

The experimental results were used to develop a process flow diagram, flow-sheet and cost estimation models of a scaled-up, semi-continuous version of the sCO_2_ whey protein fractionation process. WPI at a rate of 200 pounds per hour (90.7 kg/h) mixed with water to form a solution of 10% or 5% WPI concentration, was used as the incoming feed, and two enriched whey protein powders as the final products.

A flow-sheet of the various streams at different points of the process was developed in agreement with experimental results and simple mass balances with Microsoft Excel software.

The SuperproDesigner® (Intelligen, Inc., Scotch Plains, NJ, USA) computer process simulation and cost analysis program, together with mass-balance data from the flow-sheet, were employed to design a commercial-scale process flow diagram, characterize the various process flows and equipment, and aid in the development of the capital and operating costs.

Capital costs were estimated with the SuperproDesigner® simulation and costing module. Costs of all major process equipment (including high-pressure reactor, high-speed centrifuge, spray-dryer, drum dryer, compressor) were based on information received from equipment suppliers and from in-house estimating sources. The total plant installed cost was calculated from total equipment costs through the use of Lang factors [45]. A total plant cost of three times the estimated cost of facilities equipment included: supply and installation of the process equipment and all support materials such as piping, electrical, instrumentation; foundations and buildings to house the equipment; facilities design and construction management; start-up expenses.

The production time was fixed as 22 hours/day, 250 days/year. Process operating costs included raw materials (WPI: $10.70/kg in July 2011), water ($0.71/m^3^), steam ($12/ton), electricity ($0.10/kWh), maintenance time (2 hours/day), maintenance cost (6% of capital cost per year), operators (two per shift, $45/hour), and insurance (1% of capital cost per year). We assumed full recycling of CO_2_.

### 2.6. Centrifugation

To choose an industrial-size centrifuge, the influence of centrifuge speed on the general compaction of α-LA aggregated particles and the retention of β-LG-rich solution between and within the particles was studied as a function of initial solution concentration (C = 5, 10 and 15wt %), due to the assumption that protein concentration will affect the viscosity of the WPI solution and thus change the viscous drag in effect during particle sedimentation. Identical 40-mL samples of sCO_2_-treated WPI (as described above) were centrifuged at 4 different speeds for 30 min. The force in the middle of the tube was chosen as the following: 2000 g, 5000 g, 10,000 g and 20,000 g (corresponding to 5000 rpm, 8000 rpm, 11,500 rpm and 16,000 rpm according to rotor specifications, respectively).

### 2.7. Foam Stability

To design the commercial-size de-foaming tank needed at the reactor exit (after depressurization of the sCO_2_-treated protein mixture), we studied some of the general foaming properties of the sCO_2_-saturated WPI solution. WPI solutions of various concentrations (20 to 150 g/L) were saturated with CO_2_ and equilibrated for 15 min at 60 °C and various values of P (4 to 31 MPa), to study the effect of solubilized-CO_2_ and protein contents on the quality of the foam. Large foam samples (500 mL) were extracted through the dip-tube and general foam attributes (initial density, liquid drainage rate and total lifetime) were recorded.

## 3. Results and Discussion

### 3.1. Pilot-Scale Fractionation Results

**Figure 2:**
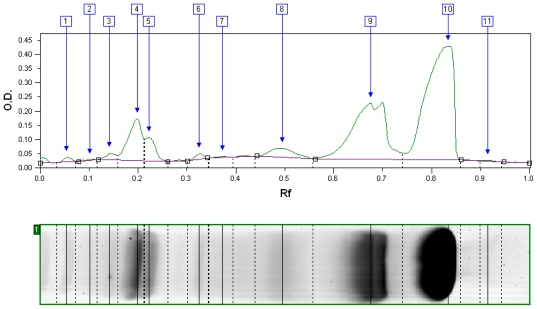
Typical sodium-dodecyl-sulfate polyacrylamide gel electrophoresis (SDS-PAGE) profile of the aggregate fraction as analyzed with densitometry. Color-density peaks are ordered by decreasing molecular weight of proteins: Peaks #1 to 6, lactoferrin, bovine serum albumin and immunoglobulins; #7, 8, 11, casein and casein fragments; #9, β-lactoglobulin (β-LG); #10, α-lactalbumin (α-LA).

The supercritical CO_2_ process effectively enabled fractionation of the proteins of WPI into a solid, α-LA-enriched fraction and a soluble, β-LG-enriched fraction, via precipitation of α-LA under acidic conditions between pH 4.4–5 at 60 to 65 °C and under CO_2_ pressures of 5.5 to 31 MPa for 2 hours. Both fractions contained no salt, acid or other contaminant after processing, and had a final pH of 6.0. Figure 2 shows the typical protein distribution of the precipitate fraction after centrifugation and drying, as measured with SDS-PAGE and analyzed with densitometry: peaks 1 through 6 correspond to lactoferrin, bovine serum albumin and immunoglobulins; peaks 7, 8 and 11 are most likely caseins and casein fragments; and peaks 9 and 10 correspond to β-LG and α-LA, respectively. The double peak for β-LG is due to the two different conformations of β-LG [13].

The initial WPI contained almost three times more β-LG than α-LA, and a small amount of minor whey proteins. After sCO_2_ processing, the precipitate fraction was typically comprised of mainly α-LA and most of the minor whey proteins, with a non-negligible amount of co-precipitated or entrapped β-LG. On the other hand, gel electrophoresis results for the soluble fraction showed mostly β-LG, with a small amount of non-precipitated α-LA and little to no minor whey proteins. GPM did not appear on the gels.

**Figure 3:**
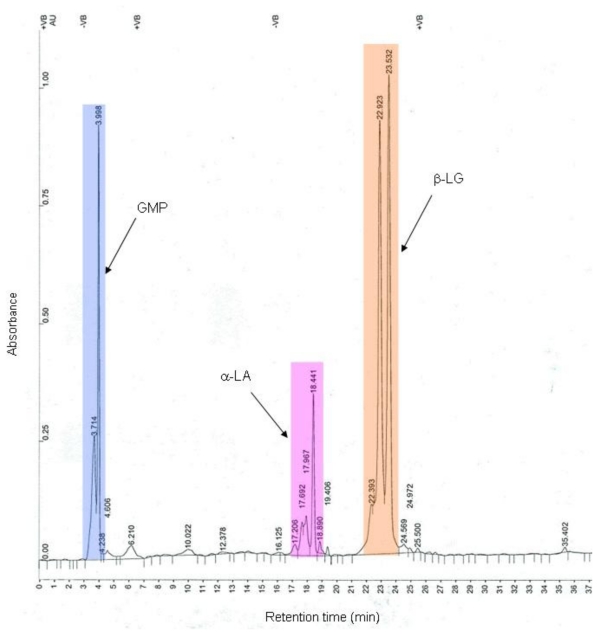
Typical high-pressure liquid chromatography (HPLC) chart of the soluble fraction after sCO_2_ treatment.

The presence of large quantities of GMP in both the initial WPI and the soluble fraction after processing was identified with HPLC analysis, Figure 3. The retention times corresponding to the various proteins were determined using protein standards. The peaks between 3.7–4.0 min retention time were identified as GMP; the peaks for α-LA appeared between 17 and 19.5 min; the peaks corresponding to β-LG typically appeared between 22 and 24 min; and all the smaller peaks between 4.5 and 30 min were attributed to BSA, Ig, Lf or casein according to their standards.

Prior results found that the total amount of precipitated proteins and the relative proportions of all proteins in the two fractions greatly depend on the process parameters (time, C_WPI_, T, and P) that influence the aggregation and precipitation kinetics of the different whey proteins, as described in detail by Bonnaillie and Tomasula [14]. For example, increasing the fractionation temperature, T, at constant pH and WPI concentration (*i.e.*, constant P and C_WPI_) considerably accelerated the precipitation rates of α-LA, β-LG, and the minor whey proteins out of the WPI solution. However, the individual aggregation rates and behaviors greatly depended on the values of T, C_WPI_ and pH. At all concentrations between pH 5.0 and 4.2, the precipitation of α-LA accelerated with T between 55 and 64 °C, and was maximized when T ≥ 64 °C, the thermal denaturation temperature of α-LA [46]. On the other hand, precipitation of β-LG increased slowly between 55 ≤ T ≤ 62 °C, then accelerated significantly when T ≥ 65 °C. Consequently, at all pressures between 8 and 31 MPa, sCO_2_ treatment of WPI solutions between 60–62 °C produced moderate amounts of fairly pure α-LA precipitate, depending on pH, while processing at or above 65 °C produced larger amounts of precipitate with lower α-LA purity due to greater β-LG contents. The introduction of β-LG in the precipitate fraction was found to be two-fold: a portion of β-LG was entrapped within the α-LA aggregates due to water-holding of the β-LG-rich solution by the very hydrophilic α-LA aggregates [47], while another part of the β-LG precipitated due to thermal- and pH-driven denaturation and/or pressure or anti-solvent effects of CO_2_. A study comparing the precipitation of β-LG with hydrochloric acid (HCl) and sCO_2_ to differentiate pH and pressure/anti-solvent effects is underway in our laboratory.

WPI fractionation studies with HCl showed that the optimal combination of α-LA purity and yield in the precipitate fraction was obtained at pH 4.0–4.2, T = 60–62 °C, and a long residence time (t ≥ 1 hour). Experimental runs with the sCO_2_ process were performed near the assumed range of optimal conditions: T = 60 to 65 °C, C_WPI_ = 5 or 10%, and P = 5.5, 8.3 or 31 MPa (corresponding to pH 5.0 to 4.4 depending on C_WPI_). P = 8.3 and 31 MPa were chosen as low and high pressure values in the supercritical range, limited by the reactor’s maximum working pressure of 34 MPa. P = 5.5 MPa is the pressure of the liquid CO_2_ tank at room temperature, and was added later as described below. Different combinations of parameters were used to vary recovery of the various proteins and produce fractions with different purities and yields, to analyze the relationship between processing costs and products quality and quantity. Table 1 presents the series of experimental conditions tested, with residence times of 2 hours for each run.

Table 1 shows that both the rates of precipitation of α-LA and β-LG are highly sensitive to temperature, WPI concentration and pH (C, P), which renders optimization of the sCO_2_ fractionation process complex due to conflicting parametric effects on the purities and yields of the products, as well as on the economy of the process, as studied below.

In the range of processing conditions tested, purity of the solid α-LA fraction varied from ~39% to 61% (w/w), a two- to three-fold enrichment compared to the initial WPI, with α-LA recovery between ~60–98% in the solid fraction, and β-LG and GMP recoveries of ~63–89% and ~88–97% in the liquid fraction, respectively. The yield of solid fraction varied between ~21% and 40% of the initial WPI powder. To simultaneously compare the effect of processing parameters on both the precipitation rates of α-LA and β-LG, a ‘purity ratio’ was introduced, defined as the ratio of α-LA recovery over β-LG recovery in the solid fraction:

(4) $$\text{Purity ratio}={\text{rec}}_{\alpha -\text{LA}}/{\text{rec}}_{\beta -\text{LG}}$$

Between two sets of processing conditions, a larger value of the purity ratio signifies that the precipitation rate of α-LA was greater than that of β-LG.

At constant T and C_WPI_, the total yield of precipitate generally increased with increasing sCO_2_ pressure (*i.e.*, decreasing pH), while the relative rate of α-LA/β-LG precipitation (purity ratio) decreased with P. This means that β-LG precipitation was more positively affected by P than α-LA precipitation, producing a greater solid yield with a lower α-LA purity, together with a lower β-LG recovery in the liquid fraction. This trend is opposite to that previously observed with HCl, where the rate of α-LA precipitation increased faster than that of β-LG when pH was lowered between pH 5.0 and 4.0 [14]. Thus, pressure or anti-solvent effects of sCO_2_ may possibly be reducing the stability of β-LG in the CO_2_-saturated WPI solution. According to these results, lower pressure experimental runs were added using the tank’s pressure of 5.5 MPa, with C_WPI_ = 10% and T = 60, 62 and 65 °C. Although out of the supercritical region, α-LA and β-LG followed the same precipitation trends, and α-LA fractions with a greater purity were obtained at all values of T.

At constant C_WPI_ and P, increasing T from 60 to 65 °C greatly increased the recovery of both α-LA and β-LG in the solid fraction in all cases, resulting in a much greater solid yield. However, the purity ratio was reduced considerably because the precipitation of β-LG accelerated faster than that of α-LA. From 60 to 62 °C (rows 1 and 6), the rate of α-LA precipitation increased noticeably, while β-LG precipitation was almost unchanged, resulting in both a higher purity ratio and a slightly higher solid yield.

Thirdly, reducing C_WPI_ at constant T and P had a null or negative effect on the recovery of β-LG in the solid fraction, mostly due to lesser β-LG entrapment by water-holding, and its effect on α-LA precipitation varied according to pH. The highest α-LA fraction purity, 61%, was obtained with C_WPI_ = 5% at 60 °C and P = 8.3 MPa (pH 4.7).

Because both the products quantity and quality were highly sensitive to the processing parameters, with conflicting trends, process optimization in terms of combined ‘yield & purity’ was not reached. The process was modeled using experimental results to calculate production costs as a function of the processing parameters, products yields, and purities, to identify feasibility and the most potentially profitable operating conditions.

### 3.2. Design and Modeling of a Semi-Continuous, Commercial-Scale Process

**Figure 4:**
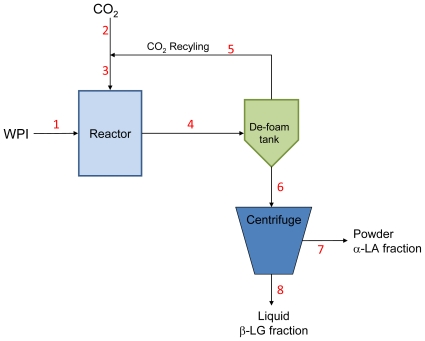
Simplified flow chart for the continuous sCO_2_ whey protein fractionation process.

Figure 4 presents a flow chart of a simplified continuous version of the sCO_2_ whey protein fractionation process, including the following base equipment and material streams: a high-pressure stirred reactor to contact the WPI solution with sCO_2_; a de-foaming tank to receive and break the foam that forms during depressurization to atmospheric pressure; a recycling loop to bring the CO_2_ that escapes from the foam back to the reactor; a high-speed centrifuge to separate the α-LA particles from the β-LG-rich solution.

To calculate all the material flows, a medium-size process was modeled with a total production rate of 90.7 kg per hour (200 lb/h) for the dried fractions, corresponding to a feed solution (stream 1) of 90.7 kg/h WPI mixed with 816 kg/h water when C_WPI_ = 10%, or 90.7 kg/h WPI mixed with 1723.6 kg/h water when C_WPI_ = 5%. Material flow rates in streams 6, 7 and 8 were calculated with mass balances using the experimental individual protein recoveries derived from the SDS-PAGE and HPLC compositional analyses. Material flow rates in streams 2, 3, 4 and 5 were calculated using experimental data for the solubility of CO_2_ in WPI solutions, and assuming complete vaporization, removal, and recycling of the dissolved CO_2_. During extraction from the reactor and depressurization down to atmospheric pressure, dissolved CO_2_ and whey proteins form a foam, which collapses in the de-foaming tank. We assumed full CO_2_ recovery from the headspace of the de-foaming tank for recycling purposes. In reality, a small percentage of CO_2_ remains solubilized after foam collapse, as evidenced by the difference between pH measured immediately after foam collapse, pH 6.0, and pH values up to 6.8 (depending on C_WPI_) after the remaining CO_2_ had time to diffuse out of the fractions (1–2 days). This non-recyclable portion of CO_2_ was neglected in mass balance calculations. At operating conditions of 8.3 MPa and a 907 kg/h feed, an ideal CO_2_ recycling loop would return 47 kg/h of CO_2_ (~23,500 L/h at atmospheric pressure) to the reactor. Stream 2 serves only in the transient phase (initial CO_2_ charge of reactor); once the process has reached steady-state, CO_2_ circulates within the recycling loop only (streams 3, 4, 5).

Table 2 presents the material flow rates in streams 1 through 8 as calculated using the experimental data with C = 5%, T = 60ºC and P=31 MPa and assuming ideal CO_2_ recycling. The partition between streams 7 and 8 in the centrifuge was calculated from the experimental recovery percentages of water and of each individual protein in the two fractions.

Mass balance calculations as presented in Table 2 were done at all values of T, P and C_WPI_ and all the calculated compositions (streams 7 and 8) matched the experimental values well and validated the accuracy of the SDS-PAGE and HPLC analyses.

#### 3.2.1. Design of the Industrial Centrifuge

We showed that the purity of the α-LA aggregate fraction depends on the efficacy of the separation between the solid and liquid fractions performed by the centrifuge. As the centrifugal force was increased from 2000 *g* to 20,000 *g*, we observed a greater compaction of the aggregate particles, resulting in reduced entrapment of β-LG-rich solution between the α-LA particles, which led to both a lower β-LG content in the α-LA fraction (*i.e.*, higher α-LA purity), and a lower total α-LA fraction yield. In theory, the sedimentation rate of the aggregate particles decreases with solution viscosity, which increases with WPI concentration. With C_WPI_ = 5% and 10%, solid compaction increased with centrifugal force between 2000 and 10,000 *g*, then reached a plateau due to the water-holding properties of whey proteins, that prevented further water removal [47]. With C_WPI_ = 15%, a plateau was not reached by forces up to 20,000 *g*. 8000 to 10,000 *g* was chosen as an optimal centrifugal force to separate the solid and liquid fractions from 5% and 10% WPI solutions and maximize both the purity of the solid α-LA fraction and the recovery of β-LG and GMP in the liquid fraction. High-speed industrial centrifuges with 8000 and 10000 *g* centrifugal forces were quoted for input in the economic simulation model (Tetra Centri D618 and D918, Tetra Pak, Vernon Hills, IL).

#### 3.2.2. Design of the De-Foaming Tank

Foam stability experiments were used to determine the density and lifetime of the whey protein foam extracted from the reactor as a function of operating CO_2_ pressure, and to calculate the size of the blending tank needed to break the foam. Between 3 and 14 MPa, both the initial foam density and rate of liquid drainage appeared approximately constant, with d_foam_~0.1 g/mL and r_drainage_~0.4 mL/min/mL_solution_, respectively. For P > 14 MPa, foam collapsed during extraction from the assumed combination of excessive shear and excessive cell-size due to the large ΔP. Consequently, at operating pressures higher than 14 MPa, a de-foaming tank is not necessary. Below 14 MPa, the volume of the de-foaming tank, −V_tank_, was calculated using Equation 5:

(5) $$V_{tank}=\frac{W_{liq}}{d_{foam}\times r_{drainage}}$$

where W_liq_ = 907 kg/h, the mass flow rate of foam; r_drainage_ = 0.4 L/min/L_solution_, the drainage rate per volume of initial liquid, and d_foam_ = 0.1 kg/L, the density of the foam. A minimum tank volume of 377 L was obtained, with a residence time of 25 minutes according to Equation 6:

(6) $$t_{s}=\frac{1}{r_{drainage}\times \frac{d_{foam}}{d_{liq}}}$$

#### 3.2.3. Process Simulation and Economic Analysis *vs.* Operating Conditions

**Figure 5:**
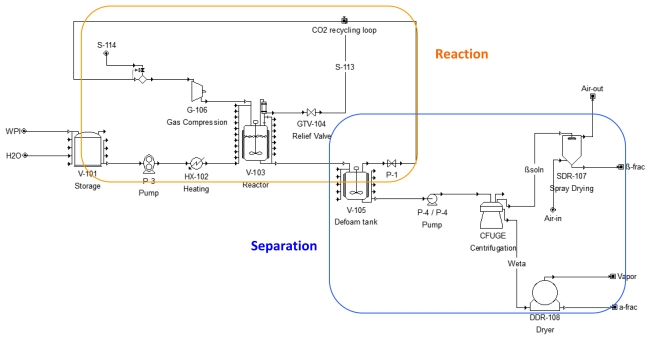
Flow-sheet of the commercial-scale, semi-continuous process drawn with SuperproDesigner®.

The commercial size process was designed as shown on Figure 5. The nomenclature and equipment used in the flow-sheet are listed in Table 3 with the cost and specification of each unit. Size, cost, main features and utility consumption of each piece of equipment was determined according to the operating conditions, namely feed and product mass and volumetric flow rates, required temperature and CO_2_ pressure in the reactor, and solid-to-water ratios in the various streams.

To simplify process design, a de-foaming tank was added in all cases regardless of sCO_2_ pressure in the reactor (Blending tank, V-105). The residence time in the tank was approximated to 30 min and the tank volume adjusted accordingly.

At constant WPI flow rate, varying the process operating conditions as described above greatly affected the capital cost of some pieces of equipment, as well as the rates of consumption of various utilities, changing the process annual operating costs considerably. The total processing costs per pound of WPI treated (not including the cost of WPI itself) as a function of the operating parameters, T, P, and C_WPI_, are listed in Table 4 for operating conditions that produced α-LA fractions with purity >50%. The corresponding experimental values of α-LA purity, recovery, and total fraction yield as obtained from a WPI with an initial α-LA purity of ~20% are listed. The respective yield of the β-LG fraction can be obtained from 1-(α-LA yield).

The total operating costs per kilogram of dry WPI treated (not including the cost of WPI itself) ranged from $5.42/kg to $12.38/kg. The lowest calculated production costs were obtained at the conditions of lowest operating pressure and highest solution concentration (P = 5.5 MPa, T = 62 °C and C_WPI_ = 10%, Case #6). In this configuration, costs were shared between facility (1,850 K$/yr), labor (540 K$/yr) and utilities (314 K$/yr), as described in ‘materials & methods’. The total production costs were distributed between the ‘Reaction’ and ‘Separation’ sections (Figure 5) to study economic trends as a function of processing conditions. The reaction section, accounting for 46 to 66.5% of total operating costs (1.3 to 4.1 M$/yr), included storage and pre-conditioning of WPI, water and CO_2_; one or two high-pressure stirred reactors; CO_2_ recycling loop; pumps, valves, compressor, heat exchangers, tubing, *etc*. The separation section (1.4 to 2.1 M$/yr) was comprised of the de-foaming tank; highspeed centrifuge; spray dryer; drum dryer; and pumps, tubing and accessories (Figure 5).

#### 3.2.4. Effects of Operating Conditions on Production Costs

Case #6 produced 0.224 kg α-LA powder with 57% purity, and 0.776 kg β-LG/GMP mixture for $5.42 per kilogram of WPI processed at 62 °C and pH 5. Raising T at constant pH to improve α-LA precipitation (case #7) adversely affected α-LA purity and raised production costs by a few cents. The additional cost was due mostly to a 40% increase in the cost of the drum dryer to dry the extra α-LA powder, while all utilities and other equipment remain mostly unchanged. The same phenomenon applies to cases 1 and 3, where α-LA yield improved, while purity and cost suffered slightly with raised T.

On the other hand, raising P to supercritical values up to 31 MPa improved yield and purity considerably (cases #4 and #5) but affected the sCO_2_ process operating costs greatly, by increasing the cost of the reactor by up to 280%, electricity and glycol consumption (for the compressor) by up to 18% and 36% respectively, and driving the total reaction costs from 1.3 to up to 2.4 M$/yr (an 86% increase).

Processing WPI feed solutions with a lower concentration at constant T and P (comparing cases #1 with #4, and #2 with #5) improved both α-LA yield and purity (up to 61%) by lowering pH and reducing β-LG entrapment in the precipitate fraction, but had the highest operating costs due to the doubling of the feed flow-rate processed. Consumption of all utilities was approximately doubled; reactor prices were doubled due to the installation of two reactors in parallel (more cost effective than one large reactor); and a larger centrifuge (52% cost increase), spray dryer (59% cost increase) and blending tank (10% cost increase) were necessary. The total reaction and separation costs were raised by up to 71% and 48%, respectively.

## 4. Conclusions

We found that supercritical carbon dioxide is an effective acid to fractionate concentrated WPI solutions into two enriched protein powders via selective precipitation of α-LA. Recovery of α-LA and total precipitate yield increased with temperature and CO_2_ pressure between 60–65 °C and up to 31 MPa. α-LA purity up to 61% was obtained from a commercial WPI containing ~18% α-LA initially, and α-LA yields as high as 40% were produced, with lower fractions purities. Operating conditions that simultaneously maximize both yield and purity were not found, thus processing parameters must be chosen according to the desired product attributes, for example: high α-LA yields with 44–55% purity, coupled with a highly purified β-LG/GMP fraction; or, smaller α-LA yields with 57–61% purity, together with higher β-LG recovery in the β-LG fraction.

Process economic simulations with state-of-the-art software SuperproDesigner® showed that with total CO_2_ recycling, this environmentally-friendly process is also economical, with production costs as low as $5.42/kg of WPI fractionated to manufacture α-LA with 57% purity and 71% recovery; or $5.52/kg for ~53% purity and 87% recovery; or $8.63/kg for 61% purity and 80% recovery. One must add the current cost of the WPI raw material ($10.70/kg at the time of this study) to the production costs to derive final prices for the α-LA and β-LG products.

Because it appeared that lower CO_2_ pressures may produce higher α-LA purity (with α-LA yield expected to decrease considerably), as well as lower equipment costs in both the reaction and separation sections, future work will explore fractionation pressures in the non-supercritical range of 3–6 MPa. The α-LA and β-LG enriched protein powders produced by carbon dioxide treatment are free of contaminants and ready for consumption. Enriched α-LA powders may be used in specialty foods and beverages targeting the nutrition of infants and the elderly, while enriched β-LG has excellent gelling properties, and is fully-soluble and ideal for protein fortification of many types of beverages. The β-LG/GMP fraction can also be further fractionated with sCO_2_ at 75–80 °C and 31 MPa to produce a purified β-LG powder and a purified GMP solution [42], useful for their valuable nutritional and nutraceutical properties in various food and drink applications.

## Figures and Tables

**Table 1:** Experimental results for the fractionation of whey protein isolate whey protein isolate (WPI) solutions with sCO_2_ into an α-LA-enriched solid fraction and a β-LG/ glycomacropeptide (GMP)-enriched liquid fraction, at different T, C_WPI_, and P. All percentages are (w/w).

							Composition solid fraction								Composition liquid fraction				

*#*	T (°C)	C_WPI_ %	P (MPa)	pH	Solid yield %	error * %	α- LA %	error * %	β- LG %	GMP %	minor %	α-LA recovery %	error * %	Purity ratio **	β- LG %	β-LG recovery %	error * %	GMP %	GMP recovery %
*1*	60	10	5.5	5.0	**21.1**^a^	1.7	**53.9**	1.5	28.0	5.5	12.6	63.3 a	6.8	**5.3**	-	88.0^a^	0.9	-	92.8
*2*	60	10	8.3	4.9	**20.9**^a^	2.5	**51.9**	2.1	28.2	4.2	15.8	60.1 a	9.5	**5.1**	68.1	88.1^a^	1.2	22.0	94.8
*3*	60	10	31.0	4.6	**28.9**	0.8	**53.9**	1.6	30.3	3.6	12.1	86.6	4.3	**4.9**	69.5	82.3	0.6	23.5	93.6
*4*	60	5	8.3	4.7	**23.6**	0.5	**61.0**	2.7	24.0	2.2	12.9	80.1	5.1	**7.0**	-	88.6	0.7	-	96.8
*5*	60	5	31.0	4.4	**28.3**	0.3	**55.5**	0.8	28.6	2.0	14.0	87.3	2.2	**5.3**	70.2	83.7	0.8	24.5	96.6
*6*	62	10	5.5	5.0	**22.4**	0.6	**57.0**	2.7	25.0	3.8	14.2	71.1	4.8	**6.3**	68.9	88.7	1.8	23.1	94.7
*7*	65	10	5.5	5.0	**29.7**	1.5	**52.9**	2.5	34.4	6.4	6.3	87.4 b	0.2	**4.2**	-	79.3	2.3	-	88.3
*8*	65	10	8.3	4.9	**35.0**	0.1	**44.7**	3.3	38.8	4.4	12.1	86.9 b	6.6	**3.2**	-	72.6	0.0	-	91.4
*9*	65	10	31.0	4.6	**40.4**	4.3	**43.5**	1.7	45.2	3.5	7.8	97.7	7.7	**2.7**	65.0	63.2	5.6	30.2	89.7
*10*	65	5	8.3	4.7	**29.3**	1.3	**51.9**	2.3	35.0	2.1	11.0	84.3 c	0.1	**4.1**	-	79.3	2.8	-	96.2
*11*	65	5	31.0	4.4	**36.5**	2.2	**38.8**	1.1	49.4	2.3	9.5	78.7^c^	6.5	**2.2**	64.1	63.6	2.7	28.7	95.3

* “error” is the standard deviation between experiment repeats;

** “Purity ratio” is defined in Equation 4;

a, b, c Not significantly different.

**Table 2:** Sample material flow rates and products compositions for the simplified process diagram of Figure 4, according to stream numbers, calculated via mass balance using the experimental data obtained at C = 5%, T = 60 °C and P = 31 MPa.

Flow rates (kg/h)	1	2	3	4	5	6	7	8
Water	864.82	0	0	864.82	0	864.82	29.40	835.42
Minerals	0.91	0	0	0.91	0	0.91	0.03	0.88
Lactose	0.45	0	0	0.45	0	0.45	0.02	0.44
α-LA	8.16	0	0	8.16	0	8.16	7.13	1.04
β-LG	22.45	0	0	22.45	0	22.45	3.66	18.79
GMP	7.35	0	0	7.35	0	7.35	0.25	7.10
Minor proteins	2.86	0	0	2.86	0	2.86	1.69	1.17
CO_2_	0.00	197.52	300.79	103.26	103.26	0	0.00	0.00
Total	907.00	197.52	300.79	1010.26	103.26	907.00	42.17	864.83

Dry composition (w/w)	1	2	3	4	5	6	7	8
Minerals	2.2%						0.2%	3.0%
Lactose	1.1%						0.1%	1.5%
**α-LA**	**19.4%**						**55.8%**	**3.5%**
**β-LG**	**53.2%**						**28.7%**	**63.9%**
**GMP**	**17.4%**						**2.0%**	**24.1%**
Minor proteins	6.8%						13.2%	4.0%
CO_2_	0.0%						0.0%	0.0%
Total	100.0%						100.0%	100.0%

**Table 3:** Equipment nomenclature and specifications for the fractionation of WPI with sCO_2_ at T = 62 °C, P = 5.5 MPa, C = 10% and a residence time of 2 hours.

Name	Description	Specification	Cost (K$)
V-101	Flat Bottom Tank	Volume = 1006.5	L 28
P-4	Centrifugal Pump	Power = 0.04	kW 9
P-3	Gear Pump	Power = 0.12 kW	1
V-103	Stirred Reactor	Volume = 2.05 m^3^	1094
HX-102	Heat Exchanger	Area = 0.15 m^2^	1
CFUGE	Disk-Stack Centrifuge	Throughput = 919 L/h	450
SDR-107	Spray Dryer	Volume = 7.61 m^3^	897
DDR-108	Drum Dryer	Area = 1.36 m^2^	370
G-106	Centrifugal Compressor	Power = 99.8	HP 400
V-105	Blending Tank	Volume = 0.46 m^3^	169
	Unlisted Equipment		603
	TOTAL		4022

**Table 4:** Total production cost per pound of WPI treated, and purity and yield of the α-LA fraction, as a function of operating parameters for a WPI feed rate of 90.7 kg/h (200 lb/h).

*Case #*	C_WPI_ (wt %)	T (°C)	P (MPa)	pH	α-LA yield (wt %)	α-LA purity	α-LA recovery	Process cost ($/kg WPI)
*1*	5	60	8.3	4.7	23.6%	61.0%	80.1%	8.65
*2*	5	60	31.0	4.4	28.3%	55.5%	87.3%	12.38
*3*	5	65	8.3	4.7	29.3%	51.9%	84.3%	8.67
*4*	10	60	8.3	4.9	20.9%	51.9%	60.1%	5.72
*5*	10	60	31.0	4.6	28.9%	53.9%	86.6%	7.65
*6*	10	62	5.5	5.0	22.4%	57.0%	71.1%	5.42
*7*	10	65	5.5	5.0	29.7%	52.9%	87.4%	5.52
